# Automated Quantification of Movement Qualities in the Human Upper Extremity After Stroke Using a Wearable Robot

**DOI:** 10.21203/rs.3.rs-8779514/v1

**Published:** 2026-02-16

**Authors:** Yoon No Gregory Hong, Kyoungsoon Kim, Sheng Li, Marcia K. O’Malley, Ashish D. Deshpande, Jinsook Roh

**Affiliations:** University of Houston; University of Houston; University of Texas Health Science Center at Houston; Rice University; University of Texas at Austin; University of Houston

## Abstract

**Background:**

Stroke is a leading cause of long-term adult disability, with approximately 80% of survivors experiencing upper extremity (UE) motor impairments. Conventional tools like the Fugl-Meyer Assessment (FMA) are widely used but limited by ordinal scales and subjective visual observation. While wearable robotics offer high-resolution data, their clinical translation is hindered by a lack of standardized protocols and limited interpretability for clinical decision-making.

**Objective:**

This study aimed to develop an objective, standardized, and clinically interpretable method to quantify UE motor qualities by integrating wearable robotic technology with traditional clinical assessment tasks.

**Methods:**

Ten healthy individuals and ten stroke survivors performed seven standardized tasks (six from the FMA-UE and one additional elbow task) while wearing the HARMONY exoskeleton. We developed a “trajectory pattern similarity score” based on the root mean square error between individual joint trajectories and normative averages. Additionally, kinematic synergy analysis was performed using non-negative matrix factorization to evaluate alterations in multi-joint coordination.

**Results:**

The trajectory pattern similarity score showed a strong negative correlation with clinical FMA-UE scores (*r* = −0.93, *p* < 0.01) and demonstrated excellent test-retest reliability (ICC = 0.98). The number of identified kinematic synergies decreased significantly as motor impairment severity increased (*r* = 0.79, *p* < 0.01). Furthermore, kinematic synergy analysis provided a mechanistic explanation for reduced individual joint control. Post-stroke synergies could be explained through the merging (linear combinations of healthy kinematic patterns), preservation, or loss of healthy kinematic synergies, reflected as pathological joint coupling and loss of specific individual joint control.

**Conclusions:**

This study presents a novel, standardized assessment framework that integrates wearable robotic technology with conventional clinical tasks. By bridging the gap between objective robotic data and clinical interpretability, this approach would enable robust motor impairment assessment and intuitive phenotyping of motor characteristics to guide personalized rehabilitation strategies.

## 1 Background

Stroke is one of the leading causes of acquired long-term disability, significantly impacting the quality of life for survivors [1]. According to the American Heart Association and American Stroke Association, stroke is the most prominent cause of long-term disability in adults in the United States. Approximately 80% of stroke survivors suffer from varying degrees of motor impairments, especially in the upper extremity. Furthermore, stroke survivors often encounter difficulties in maintaining life satisfaction due to these long-term disabilities [2]. Despite advances in acute medical care and rehabilitation, up to 60% of stroke survivors retain upper extremity motor impairments six months post-stroke, indicating the need for effective assessment and intervention strategies [3].

Motor impairment and motor function after stroke are conventionally assessed using standardized clinical tools such as the Fugl-Meyer Assessment (FMA) and the Action Research Arm Test (ARAT). These assessments are widely accepted and validated for use in clinical and research settings, providing reliable information for evaluating upper limb motor impairment/function and recovery in stroke survivors [4, 5]. However, their reliance on ordinal scales and visual observation limits the ability to capture detailed or subtle information on motor impairment and function. While these tools remain valid methods for evaluating motor impairment/function, advancements in sensing and robotic technologies enable us to develop more nuanced and objective assessments, crucial for personalized medicine.

There is a growing trend toward integrating technology to enhance the accuracy, reliability, and efficiency of motor impairment/function assessments after stroke through objective, automated solutions [6–8]. Wearable robots with advanced sensors can provide objective, high-resolution temporal data on joint angles, movement quality, and other kinematic and kinetic parameters [9] These devices can capture detailed metrics, such as range of motion, joint torques, movement smoothness, and reaction times, which are often difficult to quantify accurately with traditional clinical assessments [10–13]. Furthermore, providing intuitive and interpretable results from objective data measured by wearable robotics can bridge the gap between assessment outcomes and actionable rehabilitation strategies.

Previous studies have identified significant barriers of wearable robotics in translating objective assessments from research to clinical practice, despite their demonstrated reliability and validity. The most significant barriers are the lack of standardization in robotic assessment protocols, time constraints within clinical workflows, and limited understanding of robot data for clinical decision-making [14–16]. Addressing these issues requires the development of standardized assessment methods that provide intuitive and interpretable information to enhance understanding of motor impairment/function after stroke. Integrating robotics with standardized tasks adapted from conventional clinical assessments could provide a transformative solution. This approach ensures continuity with well-established clinical practices while leveraging the strengths of robotic systems to produce objective, repeatable, and physiologically relevant data. Thus, it will lead to the provision of not only standardized clinical scores but also interpretable and actionable individual motor characteristics with greater resolution to support personalized clinical practice.

FMA assesses motor impairment by evaluating the capacity of joint movements. Many previous studies have used wearable sensor data from FMA sub-tasks to estimate FMA scores using machine learning, aiming to achieve more objective, faster, and more reliable estimates than current visual observation [17, 18]. While valuable for automation, this approach often functions as a ‘black box’ providing limited information regarding the detailed quality of joint movement and lacking direct clinical interpretability. In this study, we suggest that instead of focusing on estimating the score itself, we focus on providing clinically interpretable complementary information alongside the FMA score. By using robotic technology to quantify these underlying joint-level qualities during the FMA tasks, relative to normative data, we can more effectively translate technological advancements into actionable clinical insights.

Thus, this study aimed to quantify motor qualities of the human upper extremity after stroke in an intuitive and clinically interpretable manner by integrating wearable robotic measurements with standardized clinical assessment tasks. Importantly, rather than estimating or automating the ordinal FMA-UE score itself, our approach focuses on characterizing underlying joint-level movement qualities during FMA-based tasks relative to normative movement patterns. This framework is designed to complement, rather than replace, traditional clinical scores by providing continuous, objective, and physiologically meaningful information about movement execution and coordination.

## 2 Methods

### 2.1 Participants

Ten healthy adult participants (Age: 48.1 ± 9.2 year) with no known upper extremity injury and ten stroke survivors (Age: 57.2 ± 9.3 year) were recruited and performed the experimental protocol in this study. Stroke participants had a single unilateral ischemic or hemorrhagic stroke that occured at least six months prior to the experiment. Additionally, they were required to be free of botulinum toxin injections in the impaired upper extremity for the minimum three months preceding the study. Participants’ FMA-UE score and anthropometric data (e.g., arm length and seated height) are provided in Table 1. All participants signed an informed consent form approved by the University of Houston Institutional Review Board in accordance with the Declaration of Helsinki (STUDY00003895).

### 2.2 Equipment

**Figure 1:**
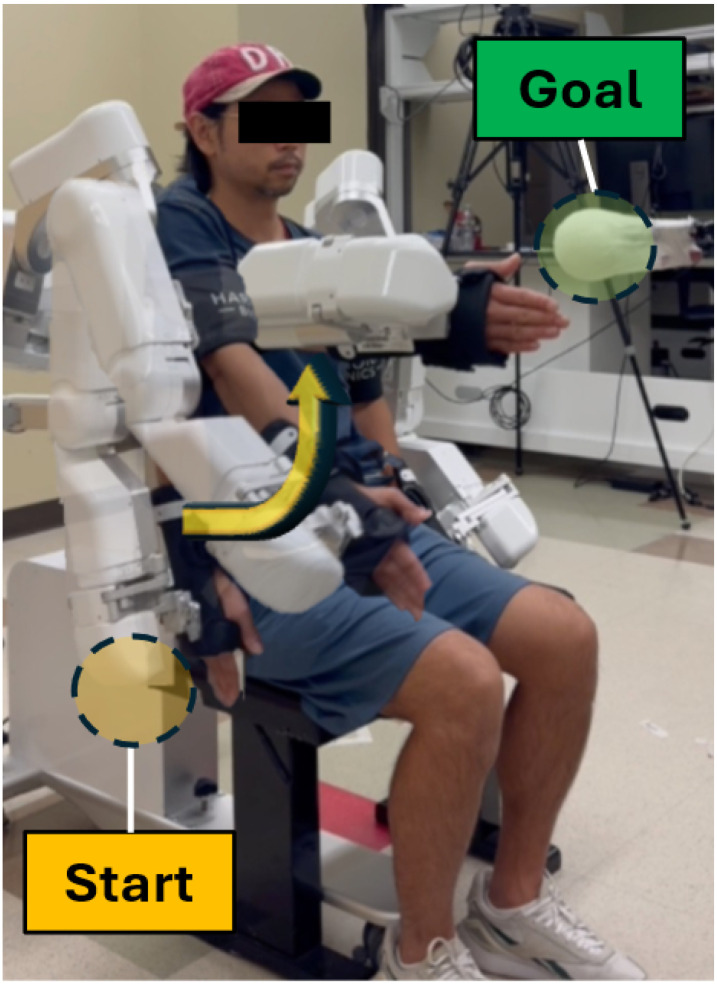
A participant seated and wore the HARMONY exoskeleton (Harmonic Bionics, Austin, TX) performing Task #2 (Shoulder Flexion at 90°; Sh_FE_90_). The HARMONY is a bilateral upper extremity exoskeleton with 7 degrees of freedom per arm, including shoulder protraction-retraction, elevation-depression, flexion-extension, abduction-adduction, internal-external rotation, elbow flexion-extension, and wrist pronation-supination. This exemplary figure demonstrates the experimental setup, where the participant is guided by physical targets—a yellow circle as the start position and a green circle as the goal. Verbal instructions and visual guidance by using these physical targets were provided as needed for each experimental condition.

HARMONY exoskeleton (Harmonic Bionics, Austin, TX, Figure. 1) was used to record the joint angle while performing sub-tasks of the FMA-UE. The HARMONY is a bilateral UE exoskeleton with 7 degrees of freedom on each side of the arm, including shoulder protraction-retraction, elevation-depression, flexion-extension, abduction-adduction, internal-external rotation, elbow flexion-extension, and wrist pronation-supination which can provide wide range of motion (RoM). In addition, The exoskeleton utilizes a baseline control strategy that actively compensates for gravitational and frictional forces, ensuring minimal resistance to the voluntary movements of participants. [19].

### 2.3 Experimental Protocol

Joint trajectories were recorded using the exoskeleton’s encoder, one multi-joint task (Task 1, Table 2) and six single-degree-of-freedom (DoF) tasks (Task 2–7, Table 2). Tasks 1–6 were adopted from the standardized FMA-UE task [20], while Task 7 was included as an extra single-DoF movement for the analysis of elbow joint movement, which is not included in the FMA-UE (Table 2). Participants performed all seven tasks for five times each in a pseudo-randomized order, while the operator provided verbal instruction as necessary (e.g., auditory cue). If some participants were unable to achieve the standardized initial posture due to severe motor impairment, the initial position was adjusted to their maximum attainable physiological limit closest to the target posture. Participants were then instructed to perform the task to their maximal voluntary extent. This approach ensured safety and comfort while preserving the inclusion of severe motor phenotypes. Two sessions were conducted in different days for the test-retest reliability evaluation. Also, physical objects indicating a start and end-point of movement were provided to ensure consistent arm movements across repetitions and sessions (Fig. 1).

### 2.4 Data Acquisition and Analysis

To record joint angles, the built-in rotary encoder at each DoF of exoskeleton was used at a sampling rate of 200Hz. The exoskeleton’s coordinate system was determined through forward kinematic analysis, using the predefined Denavit-Hartenberg (DH) parameter and the low-pass filtered sensor data (4th order Butterworth, *f*_*C*_ =10Hz).

Joint trajectory was trimmed to include only the movement phase, defined from the movement onset to termination. The movement onset was identified when joint velocity exceeded 10% of the maximum velocity, and termination was determined when velocity dropped below 10%. Lastly, the trimmed datasets were then normalized temporarily normalized to a 0–100% scale, enabling comparison across participants.

To quantify the deviations of joint trajectory patterns from normative patterns, we calculated the root mean square error between individual stroke survivors and the average joint trajectories of healthy participants. This average serves as the normative reference for each joint. In this study, the mean joint trajectories derived from age-matched healthy participants were used as a reference to represent typical movement patterns under the same experimental and task constraints. This reference was not intended to represent a universal normative database, but rather to provide a consistent baseline for quantifying relative deviations in joint trajectory patterns within the present cohort. We aggregated the joint trajectory pattern error for each task to evaluate a quality score at the task level. Subsequently, we combined all task-level quality scores to derive a total score, providing a comprehensive view of motor quality. To establish the temporal stability of the developed scoring systems, test-retest reliability was assessed over different days, with the intraclass correlation coefficient (ICC) employed as the measure of agreement [21].

To evaluate changes in joint coordination, we used kinematic synergy analysis, applying a non-negative matrix factorization (NMF) method to the joint kinematic data [22]. Given that NMF imposes positive constraints, we separated each degree of freedom of the joint into two distinct motions (e.g., shoulder internal and external rotation was treated separately). This approach reduced data dimensionality by reconstructing joint trajectories as linear combinations of kinematic synergy vectors and their corresponding activation profiles. For each potential number of kinematic synergies, ranging from one to the total number of joint degrees of freedom, we repeated the extraction of the kinematic synergies 100 times with random initial values to avoid falling into local minimum errors [23, 24]. The set of kinematic synergies that produced the highest global Variance Accounted For (gVAF) was selected as the representative set for each number of kinematic synergies. To determine the appropriate number of kinematic synergies, we evaluated how well these synergies explained the total variation in all kinematic data, aiming for a gVAF greater than 90% [25].

We also assessed whether the identified kinematic synergies in stroke survivors could be represented as a linear combination of the kinematic synergies observed in healthy age-matched individuals. This approach was based on a method from a previous study by Cheung et al [26]. The coefficients for this linear combination were determined using a standard non-negative least-squares procedure. A healthy kinematic synergy was considered to contribute to the merging if its associated coefficient exceeded 0.2. Furthermore, we evaluated whether the reconstructed kinematic synergies were significantly similar to the original kinematic synergies in stroke individuals using scalar product between the original and the corresponding reconstructed kinematic synergies

### 2.5 Statistical Analysis

Pearson correlation analysis was used to determine whether the outcome measures in this study and FMA-UE score showed a statistically significant relationship (*p* < 0.05).

## 3 Results

### 3.1 Joint trajectories of movement in healthy and stroke participants

**Figure 2:**
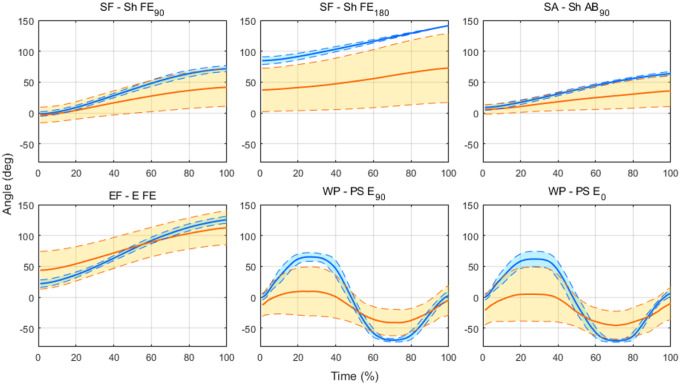
Example of joint trajectories during six single-degree-of-freedom movement tasks, corresponding to FMA subtasks. The thick, solid blue line and orange line indicate the mean trajectory of movement, performed by healthy and stroke participants, respectively. The dashed blue and orange line indicate ±1 standard deviation. SF, shoulder flexion. SA, shoulder abduction. EF, elbow flexion. WP, wrist pronation.

Figure 2 shows that joint trajectories of six single-degree-of-freedom movements healthy participants (n = 10) performed were concentrated within a narrow range, as clearly indicated by the small standard deviations. In contrast, the stroke group (n = 10) showed a reduced range of motion with a greater inter-subject variability compared to the healthy group, reflecting the broad range of severity within the stroke group. Depending on the severity levels and static posture status, some post-stroke participants could not achieve the verbally instructed initial and final postures. For example, severely impaired participants (FMA score < 25/66) were unable to reach the extreme angles for shoulder flexion and abduction. Also, stroke survivors with established joint contracture were unable to fully extend the elbow, failing to achieve the initial posture of the E FE task. The robust and consistent trajectory pattern observed in the healthy group, juxtaposed with the deviations, from the trajectory pattern of the healthy group, observed in the stroke group, suggests that a scoring system based on deviations from the mean healthy trajectory would be a reliable, intuitive, and simple method to quantify motor impairment severity.

### 3.2 Proposed metric: trajectory pattern similarity score

**Figure 3:**
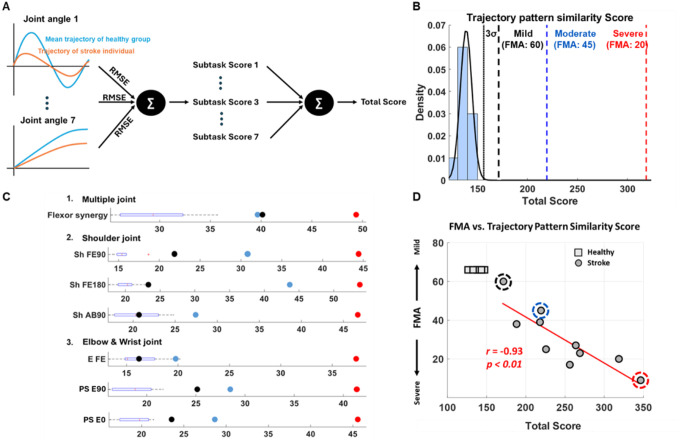
Kinematic trajectory pattern similarity score between age-matched control and stroke. **A,** Schematic diagram of calculating trajectory pattern similarity score. **B** and **C,** Healthy participants’ trajectory pattern similarity total (**B**) and subtask score distribution and exemplary score of three stroke individuals with mild (black dashed line), moderate (blue dashed line) and severe (red dashed line) motor impairments (**C**). **D,** Association between the trajectory pattern similarity score and the FMA-UE score. A strong negative correlation (r = −0.93) was observed between the trajectory pattern similarity score and the Fugl-Meyer Assessment of the upper extremity (FMA-UE) score, indicating that higher trajectory deviation (less similarity) corresponds to more severe motor impairment. Healthy participants are indicated by squares, while stroke participants are indicated by circles, with severity levels differentiated as mild (blue dashed circle), moderate (yellow dashed circle), and severe (red dashed circle).

We developed a new scoring system based on how much the joint trajectory patterns of stroke individuals deviate from the mean joint trajectory patterns of the healthy group at each task and joint (Fig. 3A). The summation of these joint-specific RMSEs across tasks yields a total score, representing the cumulative trajectory pattern similarity. As shown in the probability density function (Fig. 3B), the healthy group exhibited a highly consistent and narrow distribution (total score range: 126–147). In contrast, the stroke group’s scores were widely dispersed (total score range: 171–347), remaining outside the 3σ boundaries of healthy distribution. To provide clinical context within this distribution, three representative individuals were shown: a mildly impaired individual (FMA: 60), a moderately impaired individual (FMA: 45), and a severely impaired individual (FMA: 20). The scores for these representatives demonstrate that the scoring system effectively scales with the severity of motor impairment as categorized by traditional clinical assessment score. This scoring system also offered detailed information about specific tasks and joint levels, which were also well aligned with severity levels of motor impairment (Fig. 3C). Across tasks, the trajectory scores for stroke survivors consistently fell outside the boxplot whiskers of the healthy distribution. While the three representative individuals generally maintained their relative ordering across tasks, their relative positions shifted across different tasks; this individual heterogeneity suggests that the scoring system captures task-specific deficits unique to each stroke survivor. Moreover, the clinical validity of the Trajectory Pattern Similarity Score was confirmed through its relationship with the FMA-UE. It showed a strong correlation (r = −0.93, p < 0.01, Fig. 3D) with the FMA-UE score.

### 3.3 Test-retest reliability of score

The ICC results revealed the strong test-retest reliability for both the total score and individual subtask scores (Table 3). The total score achieved an ICC of 0.98, indicating a high degree of measurement consistency over time. Reliability for the individual subtask scores was also good to excellent with ICC values ranging from 0.87 to 0.96.

### 3.4 Typical pattern of kinematic synergies in healthy group

**Figure 4:**
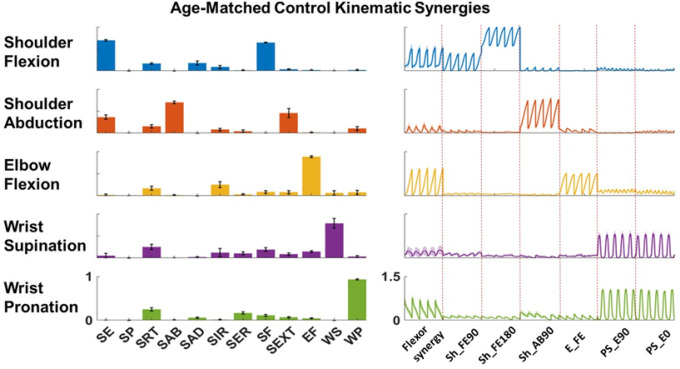
Example kinematic synergies of one typical age-matched healthy participant. The five distinct synergies captured coordinated joint movements across the upper extremity, reflecting typical motor patterns in healthy individuals. Each synergy demonstrated a dominant joint movement (e.g., shoulder flexion, shoulder abduction, elbow flexion, wrist pronation, or wrist supination), often combined with compensatory actions such as scapular elevation or joint extension, highlighting the complexity of multi-joint coordination. Per movement, five repetitions were reflected as the five peaks of each kinematic synergy activation profile.

We identified five kinematic synergies that represented shoulder flexion, shoulder abduction, elbow flexion, wrist pronation, and wrist supination in the healthy group (Fig. 4). The shoulder flexion synergy primarily consisted of scapular elevation and shoulder joint flexion movements. The shoulder abduction synergy mainly included shoulder joint abduction along with shoulder joint extension and scapular elevation. The remaining three synergies were dominated by their respective main movements. Analysis of the activation profiles confirmed that the extracted synergies were selectively recruited during their intended tasks, verifying the robustness of the synergy identification process.

### 3.5 Relationship between the number of kinematic synergies and severity of motor impairment in stroke

**Figure 5:**
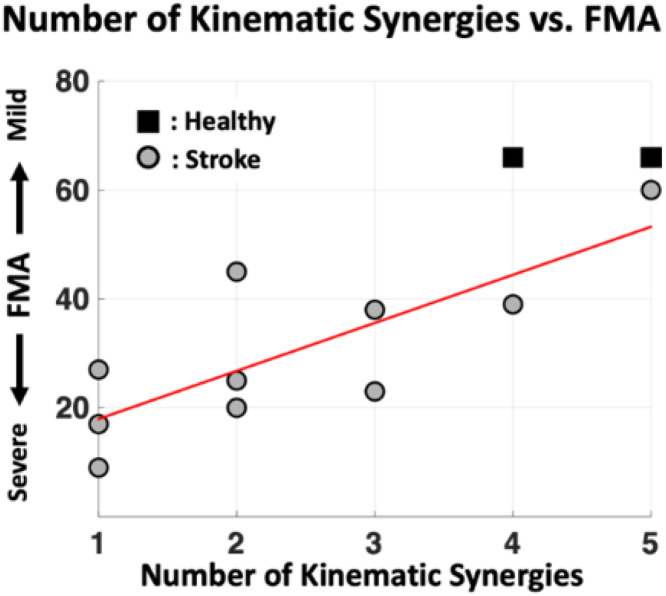
Relationship between the number of kinematic synergies and the severity of motor impairment. A positive correlation (r = 0.79, *p* < 0.01) was observed between the number of identified kinematic synergies and Fugl-Meyer Assessment (FMA) scores, suggesting that a higher number of synergies is associated with less severe motor impairment. Healthy participants are indicated with black squares, and stroke participants with gray circles.

We found that the number of kinematic synergies in stroke survivors was significantly associated with FMA-UE score (r = 0.79, *p* < 0.01, Fig. 5). The number of kinematic synergies decreased as the severity of motor impairment increased.

### 3.6 Characteristics of kinematic synergies in stroke

**Figure 6:**
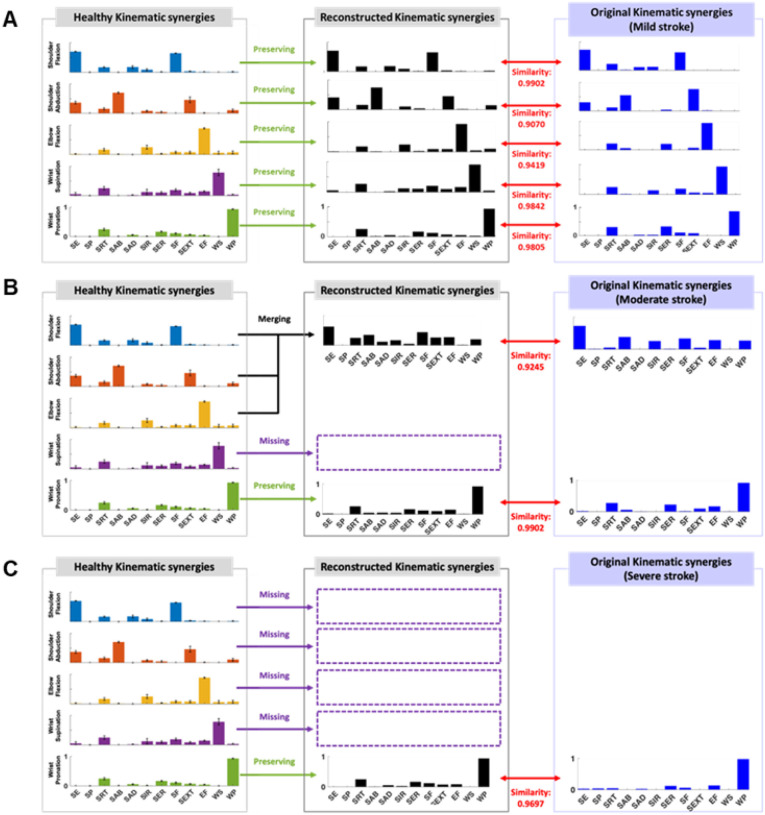
Example of reconstructed kinematic synergies in a stroke participant (**A**: Mild, **B**: Moderate, **C**: Severe) compared to age-matched healthy controls, represented in the first column. For example, a stroke survivor with moderate impairment (**B**) exhibited two kinematic synergies, characterized by a merging of shoulder flexion, shoulder abduction, and elbow flexion (blue, orange, and yellow bars) of healthy individuals, preserving wrist pronation (green), and missing wrist supination (purple). This decomposition illustrates how stroke-related synergy loss can be interpreted as a linear combination of healthy kinematic synergies, with specific features either merged, preserved, or omitted.

We characterized the reduced number of kinematic synergies in stroke survivors based on preservation, missing, and a linear combination of healthy kinematic synergies. This approach allowed us to accurately reconstruct the kinematic synergies of stroke survivors with the varying level of motor impairment. For instance, one participant with moderate impairment exhibited two kinematic synergies (Fig. 6B). When we reconstructed these synergies using a linear combination of healthy ones, the results showed a merging of shoulder flexion, shoulder abduction, and elbow flexion, while preserving wrist pronation and omitting wrist supination. The similarity between the reconstructed kinematic synergies and the originals was measured at 0.92 for the merged synergy and 0.99 for the preserved synergy (Fig. 6B).

## 4 Discussion

This study developed a new objective, automated method to assess motor qualities following a stroke by integrating wearable robotic technology with conventional, standardized clinical assessment tasks. The results indicated that the newly developed metric, kinematic trajectory pattern similarity score had a strong correlation with traditional clinical assessment scores. Furthermore, this method provides a multi-level perspective, allowing for both a comprehensive overview and detailed joint-level information. The number of kinematic synergies observed in stroke participants was also correlated with clinical assessment scores. Additionally, alterations in the composition of kinematic synergies were explained by identifying merging, preserving, and missing elements relative to patterns in healthy individuals, providing intuitive insights into joint function coupling, preservation, and deficits after stroke.

### 4.1 The trajectory pattern similarity score can be a clinically relevant, robust motor assessment metric

Our result showed a strong correlation (r = −0.93, *p* < 0.01) between the trajectory pattern similarity score and the severity of motor impairment (FMA-UE score) in stroke survivors. This score effectively differentiated between healthy controls and stroke survivors. Previous studies have developed automated FMA systems using various sensors. Kim et al., and Lee et al., reported concurrent validity scores of 0.79 and 0.99, respectively, for their methods, which utilized a depth-sensing camera [7,27]. Song et al developed a cellphone-based automated FMA system that showed a correlation of 0.88 with FMA-UE scores [28]. Our results demonstrate comparable or higher correlations than those reported in previous studies [7,27,28]. These findings suggest that our current method can effectively assess upper limb motor impairment, achieving results comparable to the FMA-UE scores measured by physical therapists.

Crucially, a reliable assessment tool must yield consistent results over time to be useful for assessing motor impairment status and tracking motor recovery. Our scoring system demonstrated excellent test-retest reliability, with an ICC of 0.98 for the total score. Considering the current approach provides continuous scale, this result is comparable to the high reliability (ICC > 0.95, [29,30]) of the clinical FMA which provides ordinal scale. Furthermore, previous review paper evaluating kinematic assessments revealed that only peak velocity among 30 kinematic metrics showed sufficient test-retest reliability evidence (ICC: 0.74 – 0.95, [31]). This suggests that the trajectory pattern similarity score would be robust against the trial-to-trial variability.

### 4.2 The newly developed scoring system provides both intuitive and detailed insights into movement patterns after stroke

The proposed method quantifies motor impairment on a continuous scale, offering a comprehensive view along with detailed information regarding specific tasks and joints. Previous automated quantification of FMA focused on estimating each item score of FMA, which is an ordinal scale [7,27,28]. The current method may overcome limitations in resolution associated with the traditional scoring system. Additionally, task-level and joint-level scores provide detailed information on the extent to which joint trajectory patterns in stroke survivors deviate from normative patterns at each task and joint. Since previous studies mostly focused on estimating FMA scores using complicated methods such as machine learning algorithms or simple classification algorithms with multiple features [7,27,28], there were no complementary aspects of traditional clinical assessment. Thus, this assessment approach is expected to enable clinicians to intuitively interpret objective data, validate their clinical observations, and perform more detailed evaluations, complementing traditional clinical assessments.

### 4.3 The capability of individual joint control diminished as the severity of motor impairment increased

Our findings revealed a significant correlation between the number of kinematic synergies and the FMA score. This indicates that a diminished individual joint control capacity is associated with the severity of motor impairment. A previous study showed that increased overlap of cortical representations for different joints contributed to the loss of independent control of shoulder and elbow joints in chronic hemiparetic stroke survivors [32]. Additionally, abnormal coordination of upper extremity joints during tasks such as reaching is indicative of impaired motor control [33,34]. In accordance with previous results, the current study suggested that the loss of independent joint control in the affected arm is a significant marker of motor deficit following a stroke.

Given that the kinematic measure is the final outcome of motor pathways, the diminished capability for individual joint control can be attributed to a confluence of underlying motor impairments, such as muscle weakness, spasticity, and abnormal intermuscular coordination. However, while muscle weakness and spasticity significantly affect post-stroke motor qualities, abnormal intermuscular coordination would be the most significant factor in motor coordination. A previous study reported that joint individuation was not significantly correlated with spasticity, and muscle strength showed a moderate correlation, but participants with weak strength were still able to control joints individually [35]. Moreover, a reduction in the number of muscle synergies and/or a higher coactivation pattern in muscle synergy composition post-stroke were associated with the severity of motor impairments [23,26,36,37]. Recent study also demonstrated that each muscle synergy was associate with corresponding kinematic synergy in different upper limb functional tasks [38]. These lines of evidence might suggest that the loss of joint individuation could be the fundamental reduction in the neural degrees of freedom available to the central nervous system. Specifically, the observed loss of individual joint control likely reflects a transition from corticospinal-driven motor control to a heavier reliance on the more primitive cortico-reticulospinal tract, which is anatomically predisposed toward the broad, multi-joint co-activations [39–43].

### 4.4 The diminished capability of individual joint control after a stroke can be explained by merging, preserving, and missing healthy kinematic synergies

We further propose a method to characterize reduced individual joint control in stroke survivors by reconstructing characteristics kinematic synergies relative to normal kinematic synergies. We identified that the reduced individual joint control capability could result from the absence of individual joint control or/and abnormal coupling of inter-joint coordination. Previous studies have typically measured inter-joint coordination in a pairwise manner, evaluating only two specific joints at a time [44–46]. In contrast, our method considers multiple degrees of freedom across several joints simultaneously, allowing for a comprehensive evaluation of the changes in joint coordination following a stroke. This approach provides a more intuitive understanding of which degrees of freedom in joint movement are coupled, preserved, or absent.

### 4.5 Limitations

This study has several limitations. First, we only utilized six subtasks from the Fugl-Meyer Assessment for the Upper Extremity (FMA-UE) due to the restricted degrees of freedom of the exoskeleton used in our research. However, the results of our concurrent validity assessments suggest that these six subtasks, along with additional elbow flexion and extension tasks, adequately represent the complete FMA-UE score. Moreover, a previous study identified six key items from the FMA-UE based on significant findings from Rasch analysis [47]. Our current protocol included these items, except for wrist flexion and extension. Additionally, we recognize that incorporating more tasks is necessary to evaluate independent joint control metrics. Several joint movements beyond the included FMA-UE subtasks were not analyzed, potentially limiting our kinematic synergy analysis and overlooking significant information about the unexamined motions. Therefore, as we include the elbow flexion task alongside the FMA subtasks, optimized assessment tasks should be evaluated and further studied to ensure a comprehensive assessment. Second, the sample size was relatively small (10 healthy participants, 10 stroke survivors), which may limit the generalizability of the findings across the full spectrum of stroke heterogeneity. However, considering the small variability of joint trajectory measures across healthy participants and inclusion of a heterogeneous range of FMA scores (from severe to mild) would ensure that the captured kinematic patterns were representative of each population. Lastly, while the cross-sectional correlation with clinical scores is strong, longitudinal studies are required to determine the sensitivity of these metrics to intervention-induced recovery.

### 4.6 Phenotyping stroke patients and its clinical implications

Stroke is a highly heterogeneous condition where identical clinical scores often mask the complex and multifaceted nature of post-stroke motor impairment. Thus, traditional aggregate scores often fail to provide the specific resolution needed for targeted treatment planning. Phenotyping patients based on clinically interpretable characteristics is crucial to resolving this ambiguity and developing tailored rehabilitation strategies. Our approach provides a hierarchical structure of abnormal joint movements, moving from multi-joint coordination down to the individual joint level. This allows for the discretization of movement characteristics into a code immediately useful to clinicians. For example, the moderate participant in Fig. 6B can be classified as C_123_ M_4_ P_5_. In this notation, ‘C’ denotes abnormal Coupling, ‘M’ denotes Missing function, and ‘P’ denotes preserved function. Therefore, the notation indicates that shoulder flexion, shoulder abduction, and elbow flexion form a pathological synergy, while wrist pronation is absent.

This succinct coding scheme is intended as an interpretive framework to summarize complex coordination patterns in a clinically intuitive manner, rather than as a prescriptive clinical decision tool. Future studies involving therapists will be required to evaluate how such representations can be integrated into clinical reasoning, treatment planning, and longitudinal tracking of motor recovery. Moreover, by utilizing standardized clinical tasks (FMA), we ensure that the extracted kinematic synergies are clinically relevant. Because the upper extremity is capable of complex and diverse movements, constraining the assessment to these optimized tasks ensures that the resulting data is both robust and directly applicable to standard clinical practice.

## Figures and Tables

**Table 1:** FMA-UE scores of individual post-stroke participants (A) and anthropometric data of age-matched and post-stroke groups (B, Mean ± SD). The anthropometric data include the mean and standard deviation of limb segment lengths, shoulder width, and seated height for age-matched control (AMC) and stroke participants (ST01–10). Measurements include left and right forearm lengths, left and right upper arm lengths, shoulder width, and seated height.

A. FMA-UE scores (out of 66)					
	ST01	ST02	ST03	ST04	ST05
FMA-UE score	27	38	60	39	9
	ST06	ST07	ST08	ST09	ST10
FMA-UE score	20	45	23	25	17
B. Anthropometric data (Mean ± SD)					
	Left Forearm (m)	Right Forearm (m)	Left Upper arm (m)	Right Upper arm (m)	Seated Height (m)
AMC	0.29 ± 0.01	0.29 ± 0.01	0.26 ± 0.01	0.27 ± 0.01	1.02 ± 0.02
Stroke	0.29 ± 0.01	0.30 ± 0.01	0.28 ± 0.01	0.28 ± 0.01	1.01 ± 0.04

**Table 2:** Description of the seven upper extremity tasks used for functional assessments. Each task targeted specific joint movements commonly evaluated in stroke rehabilitation, including multi-joint and isolated joint movements. For example, *Flexor synergy* involved moving the hand from the contralateral knee to the ipsilateral ear, while *Sh_FE90* and *Sh_FE180* assessed shoulder flexion within specified ranges. *E_FE* evaluated maximum elbow flexion-extension range. Pronation-supination tasks (*PS_E90* and *PS_E0*) were performed under fixed elbow flexion angles of 90° and 30°, respectively.

#	Task	Description
1	**Flexor synergy**	Hand from contralateral knee to ipsilateral ear
2	**Sh_FE**_**90**_	Shoulder flexion from 0° to 90°
3	**Sh_FE**_**180**_	Shoulder flexion from 90° to 180°
4	**Sh_AB**_**90**_	Shoulder abduction from 0° to 90°
5	**E_FE**	Max elbow flexion
6	**PS_E**_**90**_	Max wrist pronation-supination with fixed elbow flexion (90°)
7	**PS_E**_**0**_	Max wrist pronation-supination with fixed elbow flexion (30°)

**Table 3:** Test-retest reliability

Task	Subtask Score							Total Score
	Flexor synergy	Sh_FE_90_	Sh_FE_180_	Sh_AB_90_	E_FE	PS_E_90_	PS_E_0_	
ICC2.1 (95% CI)	0.94 (0.81–0.99)	0.96 (0.86–0.99)	0.96 (0.85–0.99)	0.87 (0.58–0.97)	0.94 (0.79–0.98)	0.93 (0.75–0.98)	0.91 (0.69–0.96)	0.98 (0.91–0.99)

## Data Availability

The data used in this study might be available from the corresponding author upon a reasonable request.
